# Stimulatory effect of *Echinacea purpurea *extract on the trafficking activity of mouse dendritic cells: revealed by genomic and proteomic analyses

**DOI:** 10.1186/1471-2164-11-612

**Published:** 2010-11-01

**Authors:** Shu-Yi Yin, Wen-Hsin Wang, Bi-Xue Wang, Kandan Aravindaram, Pei-Ing Hwang, Han-Ming Wu, Ning-Sun Yang

**Affiliations:** 1 Agricultural Biotechnology Research Center, Academia Sinica, Taipei, Taiwan; 2 Institute of Statistical Science, Academia Sinica, Taipei, Taiwan; 3 Taiwan International Graduate Program (TIGP) - Molecular and Biological Agricultural Sciences Program, Academia Sinica, Taipei, Taiwan; 4 Graduate Institute of Biotechnology, National Chung Hsing University, Taichung, Taiwan; 5 Department of Mathematics, Tamkang University, Taipei County, Taiwan

## Abstract

**Background:**

Several *Echinacea *species have been used as nutraceuticals or botanical drugs for "immunostimulation", but scientific evidence supporting their therapeutic use is still controversial. In this study, a phytocompound mixture extracted from the butanol fraction (BF) of a stem and leaf (S+L) extract of *E. purpurea *([BF/S+L/Ep]) containing stringently defined bioactive phytocompounds was obtained using standardized and published procedures. The transcriptomic and proteomic effects of this phytoextract on mouse bone marrow-derived dendritic cells (BMDCs) were analyzed using primary cultures.

**Results:**

Treatment of BMDCs with [BF/S+L/Ep] did not significantly influence the phenotypic maturation activity of dendritic cells (DCs). Affymetrix DNA microarray and bioinformatics analyses of genes differentially expressed in DCs treated with [BF/S+L/Ep] for 4 or 12 h revealed that the majority of responsive genes were related to cell adhesion or motility (*Cdh10*, *Itga6*, *Cdh1*, *Gja1 *and *Mmp8*), or were chemokines (*Cxcl2, Cxcl7) *or signaling molecules (*Nrxn1, Pkce *and *Acss1*). TRANSPATH database analyses of gene expression and related signaling pathways in treated-DCs predicted the JNK, PP2C-α, AKT, ERK1/2 or MAPKAPK pathways as the putative targets of [BF/S+L/Ep]. In parallel, proteomic analysis showed that the expressions of metabolic-, cytoskeleton- or NF-κB signaling-related proteins were regulated by treatment with [BF/S+L/Ep]. *In vitro *flow cytometry analysis of chemotaxis-related receptors and *in vivo *cell trafficking assay further showed that DCs treated with [BF/S+L/Ep] were able to migrate more effectively to peripheral lymph node and spleen tissues than DCs treated as control groups.

**Conclusion:**

Results from this study suggest that [BF/S+L/Ep] modulates DC mobility and related cellular physiology in the mouse immune system. Moreover, the signaling networks and molecules highlighted here are potential targets for nutritional or clinical application of *Echinacea *or other candidate medicinal plants.

## Background

Dendritic cells (DCs) participate in a wide spectrum of immune cell functions including antigen-presentation, phagocytic activity and T cell-mediated immunities [1]. Currently, a number of experimental approaches are being evaluated to develop DC-based immunotherapy and gene-based tumor vaccine strategies to elicit specific immunities against specific cancers (e.g., prostate cancer) or infectious diseases [2-8]. DCs can capture and take up antigens present in peripheral tissue and transport them to primary and secondary lymphoid tissue for presentation to T cells [4]. Therefore, extensive research regarding cell migration, homing, and the cellular fate of DCs in various tissue systems is considered a critical issue. Research on mouse bone marrow-derived dendritic cells (BMDCs) has shown the importance of using experimental models for research into DC-based therapeutics [5-8]. We recently reported the effect and possible application of *Echinacea purpurea *(Ep) phytocompounds as immune-modifiers for human DCs using functional genomic and proteomic approaches [9,10]. Here, we extended our study to the mouse DC system, and investigated the effects of the chemically defined Ep phyto-extracts (BF/S+L/Ep) on immunomodulatory activity and cell migration/trafficking activities of DCs under *ex vivo *and *in vivo *conditions.

Since the 1990s, the use of *Echinacea *spp. as a medicinal plant or food supplement has gained popularity in the USA and Europe [11,12], and is now even recognized in Asia (e.g., China). The possible effects of *Echinacea *spp. extracts on *in vitro *activation of macrophages, natural killer (NK) cells, and other immune cell types [13-15], and on stimulating the expression of cytokines such as tumor necrosis factor-alpha (TNF-α), interferon, interleukin-1 and interleukin-6 [16] have all been reported. *In vivo *studies showed that treatment with *Echinacea *extracts can increase white blood cell populations in the circulation system [17], and enhance phagocytosis [18]. *E. purpurea *extracts have been evaluated in clinical trials for efficiency against the common cold, but the controversial results are continuously disputed [9,10,12,19,20]. To systemically assess the immunomodulatory effects on mouse DCs *in vivo*, we employed a chemically defined *Echinacea purpurea *extract, termed [BF/S+L/Ep], containing hypoxanthine, chlorogenic acid, caffeic acid, cichoric acid, quercetin-3-*O*-rhamnosyl- (1→6)-galactoside, kaempferol-3-*O*-rhamnosyl-(1→6)-galactoside and rutin [10].

Profiling of specific and global gene expression patterns by DNA microarray analysis coupled with proteomic analysis provides a useful approach for the investigation of complex biological phenomena [21,22] as we have previously shown for immune cell systems [9,10]. In this study, we used a network knowledge-based approach to analyze genome-wide transcriptome activity *in vitro *and *in vivo*, for correlation to specific proteome activities and special functional phenotypes in mouse immature BMDCs, in response to treatment with [BF/S+L/Ep]. Our findings suggest that [BF/S+L/Ep] was able to modulate cell adhesion-, cell mobility-, cytokine- and NF-κB signaling-related activities in primary cultures of mouse DCs. *In vivo *trafficking experiments using *ex vivo*-treated DCs demonstrated that [BF/S+L/Ep] could enhance the mobility of DCs to target specific lymphoid organs in test mouse. Moreover, bioinformatics studies allowed us to predict several candidate target molecules for the future translational studies of this or other phytocompound mixtures. The significance of our findings and potential application to future studies of human DCs are discussed.

## Results

### 1. Expression of DC markers in response to treatment with [BF/S+L/Ep]

**Figure 1:**
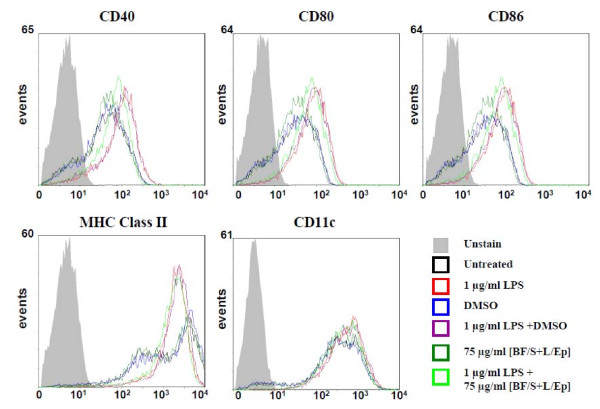
**Effects of *E. purpurea *tissue extract [BF/S+L/Ep] on expression of cell-surface markers in mouse bone marrow-derived immature dendritic cells**. DCs were examined for the expression of CD40, CD80, CD86, MHC class II and CD11c following [BF/S+L/Ep] for 24 hr by flow-cytometry.

Initially, we set out to examine whether [BF/S+L/Ep] could influence the maturation activity of BMDCs. Mouse bone marrow cells were isolated and cultured for 9 days in RPMI-1640 supplemented with 200 U/mL GM-CSF. DCs cultured in the presence of GM-CSF showed the functional and phenotypic characteristics of the immature stage and could be further differentiated *in vitro *into mature DCs. LPS, at 1 μg/mL, was then used to induce maturation of DCs. Some test cells were analyzed for the effect of [BF/S+L/Ep] for 24 h. Since concentrations of [BF/S+L/Ep] greater than 100 μg/mL were slightly cytotoxic to test dendritic cells (evaluated using MTT cell viability assays, data not shown), [BF/S+L/Ep] was used at a concentration of 75 μg/mL, where no cytotoxicity was detectable. As seen in Figure 1, sharp peak signals were obtained in flow cytometry analyses, demonstrating that high quality DCs were routinely obtained. Flow cytometry analysis on the expression of CD40, CD80, CD86, MHC class II and CD11c on BMDCs showed that the control group expressed lower levels of co-stimulatory and activation molecules than the LPS-treated groups. There was no significant difference between the DMSO-treated and [BF/S+L/Ep]-treated groups (Figure 1). Compared with the LPS group, BMDCs co-treated with 75 μg/ml [BF/S+L/Ep] and 1 μg/mL LPS only showed a weak inhibition of CD40 at 24 h post-LPS treatment, whereas expression of CD40, CD86, MHC class II and CD11c were not significantly affected. These data indicate that [BF/S+L/Ep] did not have a significant effect on the phenotypic maturation of mouse BMDCs.

### 2. Differential gene expression in immature BMDCs as a response to treatment with [BF/S+L/Ep]

**Figure 2:**
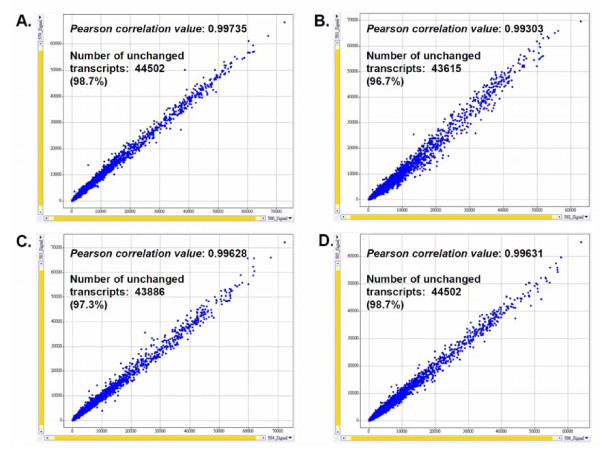
**Correlation of biological sample replicates**. **A**, DMSO (control) replicates (Affymetrix ID: 579, 580) after 4 h treatment. *Pearson correlation value*: 0.99735. **B**, [BF/S+L/Ep] replicates (Affymetrix ID: 581, 582) after 4 h treatment. *Pearson correlation value*: 0.99303. **C, **[DMSO] (control) replicates (Affymetrix ID: 583, 584) after 12 h treatment. *Pearson correlation value*: 0.99628. **D**, [BF/S+L/Ep] replicates (Affymetrix ID: 585, 586) after 12 h treatment. *Pearson correlation value*: 0.99631. Data analysis used Spotfire software. *Pearson correlation value *and number of unchanged transcripts were calculated using GCOS software.

We next investigated the expression of specific genes related to the cellular biochemistry and physiology of immature DCs. Affymetrix DNA microarray analyses showed that, although the gene expression of the cell surface markers tested above were not affected, a series of other genes related to DC activities were affected by 75 μg/ml [BF/S+L/Ep]. Initial experimental setups using biological sample replicates showed that our test cell culture systems and experimental protocols, e.g., RNA preparation, were highly reproducible and consistent, e.g., Pearson's Correlation values for test biological sample replicates were between 0.993 and 0.996 (Figure 2). All genes identified from the raw data of our Affymetrix gene chip analyses were subjected to a gene filtering proctocol, using established statistics software (Spotfire), to select and identify candidate differentially expressed genes for further studies. We chose genes that scored positive in at least 4 different chips and that had a coefficient of variation (CV) of over 0.4. A total of 907 and 1256 genes had changed expression significantly by 4 h and by 12 h post-treatment with [BF/S+L/Ep], respectively. Among these, 172 and 264 genes respectively were identified as immune-related genes (Table 1). For up-regulated or down-regulated genes, only those genes that showed at least a 2-fold change in RNA transcript level in two independent experiments were considered for further analysis. Differences in expression level were calculated by dividing the signal intensity values of genes from [BF/S+L/Ep]-treated cells by the intensity values of genes from the vehicle-treated (control) cells. Overall, similar numbers of genes were up-regulated and down-regulated after treatment with [BF/S+L/Ep] (Table 1). The expression of 29 genes more than doubled in [BF/S+L/Ep]-treated DCs, compared to vehicle control, at 4 h post-treatment, and a group of 31 genes were up-regulated (i.e., ≥ 2-fold change) at 12 h post-treatment. In contrast, 26 and 35 genes were down-regulated (expression more than halved) in [BF/S+L/Ep]-treated DCs at 4 h and 12 h post-treatment, respectively (Table 1). It is important to note that two different sets of 51 non-immune related genes were highly affected at 4 h and 12 h time points (Table 1). The relative changes in gene expression levels in [BF/S+L/Ep]-treated DCs are shown in Table 2 and Table 3 for 4 h and 12 h treatments, respectively. The unknown genes and sequences are not shown in these tables.

**Table 1:** Changes in gene expression in BMDCs in response to treatment with [BF/S+L/Ep]

	Expression change relative to DMSO alone	Up-regulated genes	Down-regulated genes	Total number of regulated genes	Percentage of all genes (%)
**At 4 h:**					
**Total genes**	Significant ^a^	513	472	985	2.90
	≥ 1.5	104	94	198	0.58
	≥ 2 ^b^	19	10	29	0.09
	≥ 3	6	5	11	0.03
	≥ 4	3	2	5	0.01
**Immune-related**	Significant ^a^	89	83	172	0.51
	≥ 1.5	16	14	30	0.09
	≥ 2 ^c^	4	0	4	0.01
	≥ 3	2	0	2	0.01
	≥ 4	0	0	0	0
**Non-immune-** **related**	≥ 2 ^b-c^	15	10	25	0.07
					
**At 12 h:**					
**Total Genes**	Significant ^a^	605	736	1341	3.94
	≥ 1.5	128	132	260	0.76
	≥ 2 ^d^	21	18	39	0.11
	≥ 3	6	5	11	0.03
	≥ 4	3	3	6	0.02
**Immune-related**	Significant ^a^	144	120	264	0.78
	≥ 1.5	27	19	46	0.14
	≥ 2 ^e^	2	0	2	0.01
	≥ 3	1	0	1	0
	≥ 4	1	0	1	0
**Non-immune-** **related**	≥ 2 ^d-e^	19	18	37	0.11

^a ^Differentially expressed genes that are significant in [BF/S+L/Ep] vs. [DMSO] (control). Values of *p *≤ 0.05 are considered to be significant. All relative differences greater than 1.5 are significant.

^b ^Up-regulated genes with at least a 2-fold change in the level of RNA transcript expression after 4 h treatment

^c ^Up-regulated immune-related genes with at least a 2-fold change after 4 h treatment

^d ^Down-regulated genes with at least a 2-fold change in the level of RNA transcript expression after 4 h treatment

^e ^Down-regulated immune-related genes with at least a 2-fold change after 12 h treatment

**Table 2:** Up-regulation of genes in iBMDCs treated with [BF/S+L/Ep] for (A) 4 h or (B) 12 h compared to DMSO alone

	Gene Name	Accession Number	**Expression Ratio**^**a **^**(+)/(-)**
**At 4 h:**			
**Cluster 1**	**Adhesion molecules, Cytoskeleton, Cell movement**		
Ncam2	neural cell adhesion molecule 2	NM_010954	6.57
Serpinb2	serine (or cysteine) peptidase inhibitor, clade B, member 2	NM_011111	4.50
Ikbkg	inhibitor of kappa B kinase gamma	NM_010547	2.03
			
**Cluster 2**	**Immune response**		
Amot	angiomotin	NM_153319	2.48
Cxcl5	chemokine (C-X-C motif) ligand 5	NM_009141	8.63
Irg1	immunoresponsive gene 1	XM_127883	2.17
Cxcl7	chemokine (C-X-C motif) ligand 7	NM_023785	2.08
			
**Cluster 3**	**Signal transmission, Transporter**		
Nrxn1	neurexin I	Q9CS84	9.62
Sorbs1	sorbin and SH3 domain containing 1	NM_009166	3.70
Ahrr	aryl-hydrocarbon receptor repressor	NM_009644	2.37
Abce1	ATP-binding cassette, sub-family E (OABP), member 1	NM_015751	2.10
			
**Cluster 4**	**Cell cycle, DNA repair, Apoptosis**		
Cyr61	cysteine rich protein 61	NM_010516	6.60
Mtap9	microtubule-associated protein 9	Q3TRR0	2.64
			
**Cluster 5**	**Enzyme, Kinase**		
Ppfia1	protein tyrosine phosphatase, receptor type, f polypeptide (PTPRF)	XM_133979	2.74
			
**Cluster 6**	**DNA binding protein, Transcription factor**		
Wdhd1	WD-repeat HMG-box DNA binding protein 1	NM_172598	2.04
			
**Cluster 7**	**Miscellaneous**		
Prdm10	PR domain containing 10	Q3UTQ7	6.98
Tdrd3	Tudor domain containing 3	NM_172605	3.48
Mif4gd	MIF4G domain containing	NM_027162	2.16
Dnahc2	dynein, axonemal, heavy chain 2	O08826	2.09
**At 12 h:**			
**Cluster 1**	**Adhesion molecules, Cytoskeleton, Cell movement**		
Serpinb2	serine (or cysteine) peptidase inhibitor, clade B, member 2	NM_011111	3.34
Cspg2	chondroitin sulfate proteoglycan 2	NM_019389	2.20
Mmp8	matrix metallopeptidase 8	NM_008611	2.18
Myo1d	Myosin ID	NM_177390	2.07
			
**Cluster 2**	**Signal transmission, Transporter**		
Phip	pleckstrin homology domain interacting protein	Q32NY1	3.28
Cd38	CD38 antigen	NM_007646	2.51
Rbp1	retinol binding protein 1, cellular	NM_011254	2.28
Ap3s2	adaptor-related protein complex 3, sigma 2 subunit	NM_009682	2.12
Golph4	golgi phosphoprotein 4	NM_175193	2.03
			
**Cluster 3**	**Enzyme, Kinase**		
Dnm1l	dynamin 1-like	NM_152816	13.11
Wdfy3	WD repeat and FYVE domain containing 3	NM_172882	4.64
Acpp	acid phosphatase, prostate	NM_019807	2.18
Apc	adenomatosis polyposis coli	NM_007462	2.15
Adhfe1	alcohol dehydrogenase, iron containing, 1	NM_175236	2.04
			
**Cluster 4**	**Immune response**		
Ctla4	cytotoxic T-lymphocyte-associated protein 4	NM_009843	11.16
Cxcl7	chemokine (C-X-C motif) ligand 7	NM_023785	2.77
			
**Cluster 5**	**RNA binding protein, Translation factor**		
Mrps5	mitochondrial ribosomal protein S5	NM_029963	2.20
Sf4	splicing factor 4	NM_027481	2.45
			
**Cluster 6**	**DNA binding protein, Transcription factor**		
Asb2	ankyrin repeat and SOCS box-containing protein 2	NM_023049	2.75
			
**Cluster 7**	**Miscellaneous**		
Dnahc2	dynein, axonemal, heavy chain 2	O08826	3.11
Ccdc89	coiled-coil domain containing 89	Q9DA73	2.01

^a ^Expression ratio is defined as relative difference in expression with (+) compared to without (-) treatment with [BF/S+L/Ep] plant extract; (-) is DMSO vehicle control.

**Table 3:** Down-regulation of genes in iBMDCs treated with [BF/S+L/Ep] for (A) 4 h or (B) 12 h compared to DMSO alone

	Gene Title	Accession Number	**Expression Ratio**^**a **^**(+)/(-)**
**At 4 h:**			
**Cluster 1**	**Adhesion molecules, Cytoskeleton, Cell movement**		
Cnn3	calponin 3, acidic	NM_028044	0.13
Cdh10	cadherin 10	Q3UUB3	0.27
Itga6	Integrin alpha 6	NM_008397	0.50
			
**Cluster 2**	**Cell cycle, DNA repair, Apoptosis**		
Sycp1	synaptonemal complex protein 1	NM_011516	0.25
			
**Cluster 3**	**Enzyme, Kinase**		
Atad1	ATPase family, AAA domain containing 1	NM_026487	0.48
St8sia6	ST8 alpha-N-acetyl-neuraminide alpha-2,8- sialyltransferase 6	NM_145838	0.45
Prkce	Protein kinase C, epsilon	NM_011104	0.47
			
**Cluster 4**	**DNA binding protein, Transcription factor**		
Shprh	SNF2 histone linker PHD RING helicase	NM_172937	0.27
Zfp39	zinc finger protein 39	NM_011758	0.31
			
**Cluster 5**	**Miscellaneous**		
Cep110	centrosomal protein 110	XM_981952	0.46
			
**At 12 h:**			
**Cluster 1**	**Adhesion molecules, cytoskeleton; cell movement**		
Cdh1	Cadherin 1	NM_009864	0.30
Gja1	gap junction membrane channel protein alpha 1	NM_010288	0.48
			
**Cluster 2**	**Signal transmission, Transporter**		
Rab17	RAB17, member RAS oncogene family	NM_008998	0.42
Oscar	osteoclast associated receptor	NM_175632	0.44
Kcne3	Voltage-gated potassium channel, Isk-related subfamily, gene 3	NM_020574	0.49
Sla	src-like adaptor	NM_009192	0.50
			
**Cluster 3**	**Enzyme, Kinase**		
Setd4	SET domain containing 4	NM_145482	0.32
Ndufb2	NADH dehydrogenase (ubiquinone) 1 beta subcomplex, 2	NM_026612	0.39
Acss1	acyl-CoA synthetase short-chain family member 1	NM_080575	0.40
			
**Cluster 4**	**DNA binding protein, Transcription factor**		
Sox6	SRY-box containing gene 6	NM_011445	0.06
Dmc1h	disrupted meiotic cDNA 1 homolog	NM_010059	0.17
Nfxl1	nuclear transcription factor, X-box binding-like 1	Q3ULV1	0.36
Huwe1	HECT, UBA and WWE domain containing 1	NM_021523	0.41
			
**Cluster 5**	**Protein metastasis**		
Fbxo36	F-box only protein 36	NM_025386	0.46
			
**Cluster 6**	**RNA binding protein, Translation factor**		
Rbm14	RNA binding motif protein 14	NM_019869	0.50
			
**Cluster 7**	**Miscellaneous**		
Speer4b	spermatogenesis associated glutamate (E)-rich protein 4b	NM_028561	0.19
Ccdc21	coiled-coil domain containing 21	NM_144527	0.47
Mgl1	macrophage galactose N-acetyl-galactosamine specific lectin 1	NM_010796	0.48

^a ^Expression ratio is defined as relative difference in expression with (+) compared to without (-) treatment with [BF/S+L/Ep] plant extract; (-) is DMSO vehicle control.

A number of genes that are reported to be differentially expressed in defined immune responses were found to have increased expression after treatment with [BF/S+L/Ep] [10]. These genes include angiomotin (*Amot*), chemokine (C-X-C motif) ligand 5 *(Cxcl5), pro-platelet basic protein *(*Cxcl7*) and immunoresponsive gene 1 *(Irg1) *which were up-regulated at 4 h post-treatment with [BF/S+L/Ep], and other genes including cytotoxic T-lymphocyte-associated protein 4 *(Ctla4) *and chemokine (C-X-C motif) ligand 7 *(Cxcl7) *were strongly up-regulated at 12 h post-treatment. Among them, *Cxcl7 *was up-regulated at both 4 h and 12 h time points (Table 2 and 3). In addition, the expression of DC surface marker genes, such as *Cd40, Cd80, Cd86, Mhc II *and *Cd11c*, showed little or no changes after [BF/S+L/Ep] treatment, confirming our previous flow cytometric analysis (Figure 1).

A number of the responsive genes found here have not previously been shown to be differentially expressed in DCs. Some of these include specific cell surface molecules related to cell adhesion or to regulation of cytoskeleton molecules, such as cadherin 10 (*Cdh10*), cadherin 1 (*Cdh1*), integrin a6 (*Itga6*), neural cell adhesion molecule 2 (*Ncam2*), microtubule-associated protein 9 (*Mtap9*), *Cd38*, and gap junction protein alpha 1 (*Gja1*). RNA transcript levels for genes encoding several secreted proteins were also increased by treatment with [BF/S+L/Ep]. These genes include chemokine (C-X-C motif) ligand 2 (*Cxcl5*), pro-platelet basic protein (chemokine (C-X-C motif) ligand 7) (*Cxcl7*), acid phosphatase (*Acpp*), chondroitin sulfate proteoglycan 2 (*Cspg2*), matrix metallopeptidase 8 (*Mmp8*), and serpin peptidase inhibitor (*Serpinb2*). In comparison, the expression of transcripts encoding several enzymes fell after treatment with [BF/S+L/Ep]. These genes included protein kinase C (*Prkce*), acyl-CoA synthetase (*Acss1*), ST8 alpha-N-acetyl-neuraminide alpha-2,8-sialyltransferase 6 (*St8sia6*), and Src-like-adaptor (*Sla*). Moreover, the expression of mRNAs encoding transcription factors or DNA binding proteins localized in the nuclear compartment, such as ankyrin repeat and SOCS box-containing 2 (*Asb2*) and inhibitor of kappa light polypeptide gene enhancer in B-cells (*Ikbkg*), were increased in [BF/S+L/Ep]-treated DCs, and the expressions other transcription regulatory genes, such as synaptonemal complex protein 1 (*Sycp1*), RNA binding motif protein 14 (*Rbm14*), HECT domain containing 1 (*Huwe1*) and SRY (sex determining region Y)-box 6 (*Sox*) were decreased (Table 2 and 3).

### 3. Putative signaling networks involved in modulatory effect of [BF/S+L/Ep] on iBMDCs

**Figure 3:**
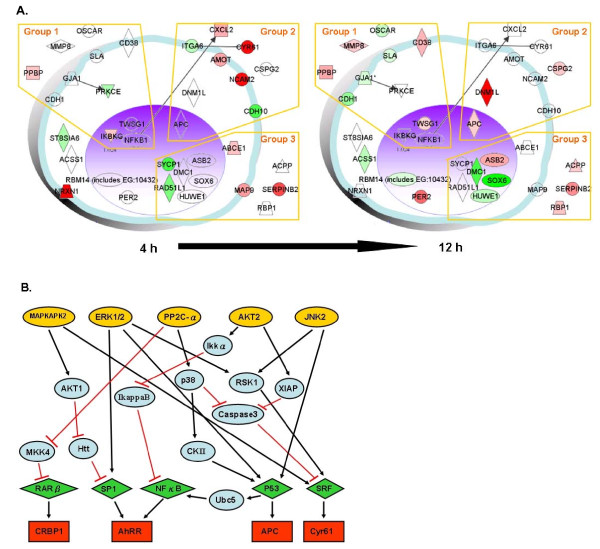
**Pathway analysis of representative genes that responded to [BF/S+L/Ep] treatment**. **A **prototypical cell was constructed from 37 representative genes that responded to treatment with [BF/S+L/Ep] *in vitro *from 4 h to 12 h. **A, **Genes whose expression was up-regulated (more than doubled) are colored red, and those whose expression was down-regulated (to less than half) are shown in green. Selected regions of the network highlight several groups of genes. Group 1, Immune response related genes. Group 2, Adhesion molecules and cytoskeleton; cell movement related genes. Group 3, Cell cycle, cell proliferation and apoptosis related genes. Gene networks were analyzed with the Ingenuity Pathways program. **B**, The TRANSPATH Professional 7.1 database was searched to assess possible signaling pathways, networks or potential interactions among the responsive genes/target molecules in DCs treated with [BF/S+L/Ep]. The 37 genes that were up- or down-regulated at least 2-fold compared to controls were analyzed. Specifically, connections (hits) within 7 genes were employed as the parameter for the current search. Five postulated key molecules/pathways; JNK2 (c-Jun NH_2_-terminal kinase 2), PP2C-α (protein phosphatase 2C-alpha), AKT (protein kinase B), ERK (extracellular signal-related kinase) and MAPKAPK (MAPK-activated protein kinase 2) that may be activated by [BF/S+L/Ep] treatment are predicted with arrows. The green rhombuses indicate the upstream transcription factors of responsive genes.

Functional genomics experimental approaches were employed in our previous study on the modulatory effect of *Echinacea *plant extracts on human DCs [9,10]. Using the same defined phytocompound extracts here we analyzed the genome-wide transcriptional response in the context of known functional activities and interrelationships among specific protein molecules and/or different cell phenotypes by using Ingenuity Systems, a structured network knowledge-based approach, to provide insight into the regulation of BMDC activities that are relevant to the body immune system. Figure 3A shows the hypothetical or candidate networks revealed by clustering analysis of representative genes involved in the BMDC response to [BF/S+L/Ep] treatment. Apparent temporal controls for coordination of specific gene expressions were classified into three different functional groups: the immune response related genes (Group 1); adhesion molecules, cytoskeleton and cell movement-related genes (Group 2); and the cell cycle, cell proliferation, and apoptosis-related genes (Group 3). These responses to treatment with [BF/S+L/Ep] extract in iBMDCs may be viewed as an integrated cell-wide response involving cell trafficking, attachment, immunity and apoptosis.

To identify possible signal transduction pathways in response to [BF/S+L/Ep] treatment, we analyzed, for both the 4 h and 12 h treatments, the 37 up-regulated genes using TRANSPATH software in the manner previously reported [9,10]. Signal transduction pathways involving the CRBP1, AhR, APC and Cyr61 genes with a ≥ 2-fold change in expression level (Table 2) were predicted. Apparent signaling network and functional genomic analyses suggest that treatment of DCs with [BF/S+L/Ep] may activate the JNK, PP2C-α, AKT, ERK1/2 or MAPKAPK pathways, because expression of their downstream genes were up-regulated (Figure 3B). For those down-regulated genes, the TRANSPATH software was not able to predict a matched upstream pathway.

### 4. Identification of differentially expressed known or novel proteins in BMDCs that respond to [BF/S+L/Ep]

**Figure 4:**
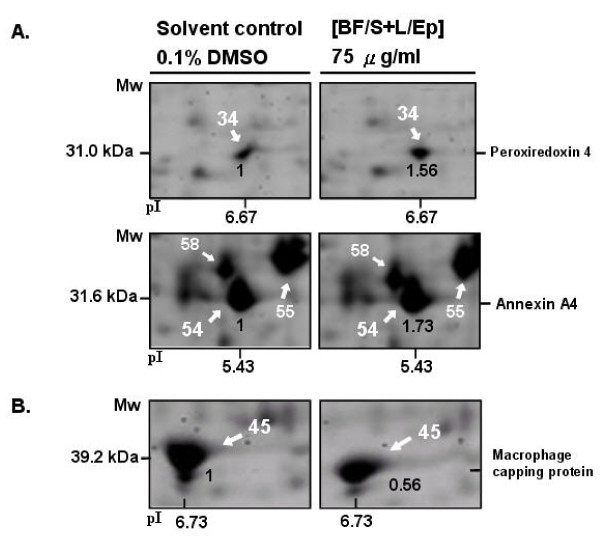
**Proteomics analyses of *E. purpurea *phytocompound effects on mouse bone-marrow derived immature dendritic cells (iBMDCs)**. Effect of *Echinacea purpurea *extract on differential protein expression in iBMDCs was evaluated by 2D gel electrophoretic analysis. 500 μg of total cell proteins from various treatments of iBMDCs were applied to pH 5-8 IPG strips. After isofocusing and 2D gel separation, SYPRO Ruby was used to stain proteins. Images on gel were analyzed using PD-Quest software. Forty-two differentially-expressed proteins were trypsin-digested *in situ*, and 23 protein spots were identified by MALDI-TOF MS, with their locations in gel assigned a number. These protein ID numbers are listed in Table 4. **A, **Up-regulated and **B, **down-regulated protein spots are reported on a representative two-dimensional gel corresponding to protein expression profile of iBMDCs. The expression ratios are shown in bold print. Gels shown here are representative of thee independent experiments.

Using 2D gel electrophoresis, we were able to routinely obtain representative, high resolution, and highly reproducible 2D protein profiles of mouse DCs as putative proteomic maps (data not shown). Treatment of DCs with [BF/S+L/Ep] at 75 μg/mL resulted in significant changes in expression of some proteins in comparison to the solvent-treated mouse DC samples. Differentially-expressed proteins were then identified by MALDI-TOF-MS and in some cases subsequently analyzed with tandem MS/MS, after manual excision of these protein spots from gels. A total of 23 different known protein species were isolated and characterized by analysis with MALDI-TOF-MS peptide mass fingerprinting (PMF). Table 4 lists the changes in protein expression in DCs treated with [BF/S+L/Ep] *in vitro *for 12 h, as compared to solvent (vehicle) treatment (0.1% DMSO). Expression of metabolism, cytoskeleton or NF-κB-signaling related proteins were most affected by [BF/S+L/Ep]. 2D gel electrophoretic analyses showed increased levels of a group of specific proteins, such as annexin A4 and peroxiredoxin (Figure 4A). In contrast, the levels of some other proteins, such as macrophage capping protein, were reduced (Figure 4B). These proteins, which function in cytoskeleton organization, did not show a detectable change in mRNA levels after [BF/S+L/Ep] treatment.

**Table 4:** Differentially expressed proteins identified by MALDI-TOF-MS/MS analysis of 2D gel protein profiles of iBMDCs treated with [BF/S+L/Ep] (75 ug/mL) in vitro for 12 h

Spot Number	Protein Name	**Relative Protein Level **^**a**^	SwissProt Accession Number	Biological Function
54	Annexin A4	1.73	P97429	Signal transduction; cell communication
34	Peroxiredoxin 4	1.56	O08807	Metabolism; Energy pathways
82	Vimentin	1.13	P20152	Cell growth/maintenance
26	Alpha-actin-2	1.12	P62739	Cell growth/maintenance
23	Proliferin-1	1.1	P04095	Cell growth/maintenance
98	APAF1-interacting protein	1.09	Q9WVQ5	apoptosis
1	beta-actin	1.05	P99041	Cell growth/maintenance
58	Alpha-tubulin 1, 2, 4, 6	1.04	P68369...	Cell growth/maintenance
79	Heat shock cognate 71 kDa protein	1.04	P63017	Protein metabolism
109	Solute carrier family 35 member E1	1.04	Q8CD26	Transport
55	Actin, cytoplasmic 1 (Beta-actin)	1.03	P60710	Cell growth/maintenance
88	YEATS domain-containing protein 4	0.96	Q9CR11	Regulation of nucleobase, nucleoside, nucleotide and nucleic acid metabolism
6	Phenylalanyl-tRNA synthetase	0.92	Q99M01	Protein metabolism
41	Protein disulfide-isomerase A3 [Precursor]	0.92	P27773	Protein metabolism
38	Phosphoglycerate mutase 1	0.87	Q9DBJ1	Metabolism; Energy pathways
78	Heat shock protein 9, [Precursor]	0.84	P38647	Protein metabolism
56	F-actin capping protein alpha subunit	0.83	P47754	Cell growth/maintenance
13	Cathepsin S	0.81	O70370	Protein metabolism
91	Nucleoside diphosphate kinase	0.74	Q9WV84	Metabolism; Energy pathways
70	Alpha-enolase	0.73	P17182	Metabolism; Energy pathways
45	Macrophage capping protein	0.63	P24452	Cell growth/maintenance
192	Voltage-gated potassium channel subfamily KQT member 2	0.46	Q9Z351	Transport
191	Protein tyrosine phosphatase mitochondrial 1	0.34	Q66GT5	signal transduction; cell communication

^a ^Relative protein level with or without [BF/S+L/Ep] (75 μg/mL), 0.1% DMSO as vehicle control.

### 5. Effect of [BF/S+L/Ep] extract on BMDC trafficking

**Figure 5:**
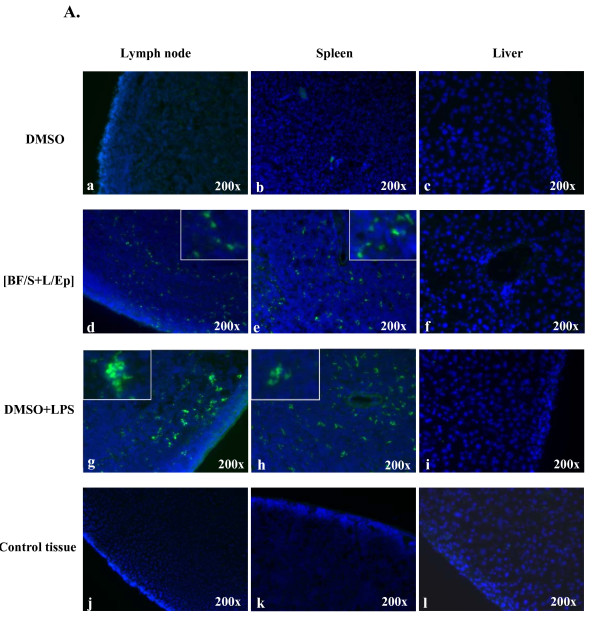
**Effects of [BF/S+L/Ep] treatment on the trafficking activity of DCs**. **A**, Two million BMDCs treated with DMSO **(a-c)**, [BF/S+L/Ep] **(d-f) **and LPS **(g-i) **were labeled with green fluorescence dye and injected into allogeneic mice. Tissues of control BALB/c mice which were injected with DCs without fluorescence dye staining did not show significant green fluorescence spots, while the auto-fluorescence background was rather low (**j-l**). After 24 h mice were sacrificed and DC mobility compared by detecting DCs with green fluorescence in peripheral lymph nodes **(a, d, g and j)**, spleen **(b, e, h and k) **and liver **(c, f, I and l)**. Insets (d, e, g and h) show digitally amplified magnifications of treated DCs exhibiting different cellular behaviors in tissue trafficking and different levels in fluorescent intensity. Samples of liver tissue were detected as negative for fluorescence staining of DCs throughout the observation period.

**Figure 6:**
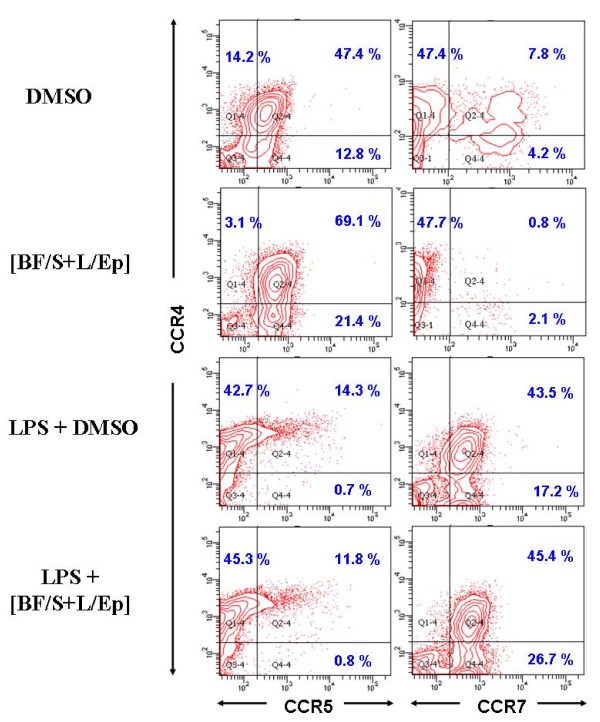
**Effect of [BF/S+L/Ep] treatment (24 h) on the expression of CCR4, CCR5 and CCR7 proteins in BMDCs treated with or without LPS stimulation**. CCR5 and CCR7 are expressed as cell surface markers on DC at distinct differentiation stages and are known to play important roles in DC mobility. CCR4^+^CCR5^+ ^and CCR4^+^CCR7^+ ^cells are considered as immature and mature DC populations, respectively. Populations of them were compared by flow cytometry analysis.

The effect of [BF/S+L/Ep] on DC mobility was evaluated by an *in vivo *DC trafficking assay. Primary BMDCs generated by *in vitro *culture were treated with 0.1% DMSO, [BF/S+L/Ep] or LPS separately. Cells were then stained with fluorescein isothiocyanate isomer I (a green fluorescence dye) and administered to test mice via i.v. injection. After 24 h, mice were sacrificed and DC motilities *in vivo *were compared by scoring the presence and number of DCs with green fluorescence dye in tissue sections of inguinal lymph node, spleen and liver. As seen in Figure 5, DCs treated with [BF/S+L/Ep] showed a higher capacity to target lymph nodes and spleen (Figure 5-d, e) than DMSO-treated DCs (Figure 5a, b; Figure 6). In comparison, DCs stimulated by LPS showed a relatively higher capacity to target these immune tissues, particularly the spleen (Figure 5-g, h). Tissues from liver of all test mice remained negative for trafficking of DCs throughout the tested experimental period (Figure 5-c, f, i and 5l). These data indicate that the Echinacea extract can stimulate enhanced migration and mobility of DCs *in vivo*, in addition to its specific effect on the transcriptome seen *in vitro*.

As shown in the insets of d and e in Figure 5, for [BF/S+L/Ep]-treated DCs, the individual cellular events of DC migration, are seen mainly as separated, single unit fluorescent dots in the photomicrographs of test tissues, and the fluorescence intensities are, in general, medium to low. In contrast, the cellular behavior and light intensities of the fluorescent test DCs in LPS-treated samples (Figure 5-g and 5h) are more aggregated and clumped together. In addition the colors are considerably brighter than those seen for the [BF/S+L/Ep]-treated DCs. It was also observed that under *in vitro *test conditions, the individual DCs treated with LPS became enlarged in size, and thus could apparently absorb higher levels of FITC-labeled dextran and also already tended to aggregate together, as compared to DCs treated with [BF/S+L/Ep]. These effects of LPS may also contribute to the observed results seen in trafficking experiments, where more aggregated and brighter DCs were detected in targeted tissues in LPS-treated DCs than in cells treated with [BF/S+L/Ep].

The potential effect of [BF/S+L/Ep] on key cellular physiological functions of DCs was further investigated in an *in vitro *study, where the expression of specific chemotaxis-related receptors for trafficking of DCs was analyzed by flow cytometry. BMDCs with or without LPS stimulation were treated with [BF/S+L/Ep] for 24 h and then assayed for expression of the three cell surface markers CCR4, CCR5 and CCR7 (Figure 6), which are known to play an important role in the mobility of DCs, are expressed on DCs at distinguishable differentiation stages, and are located at specific sites in different tissues [23]. Here, CCR4^+^CCR5^+ ^and CCR4^+^CCR7^+ ^dendritic cells were considered as immature and mature DC populations, respectively, as observed during their *in vivo *maturation processes. Our data show that *in vitro *treatment of DCs with [BF/S+L/Ep] significantly increases the expression level of CCR5 on immature DCs (Figure 6 but does not affect CCR5 expression on LPS-induced mature DCs. Furthermore, we have observed that our herbal extract treatment caused a suppressive effect on the "endogenous" or "non-LPS-induced" generation of mature DCs from immature DCs, as assessed by the CCR7 activity measured for DMSO-treated and [BF/S+L/Ep]-treated DCs (Figure 6). Considering our prior results obtained both *in vitro *(i.e., DC maturation, Figure 1) and *in vivo *(i.e., DC trafficking capacity, Figure 5), the data shown in Figure 6 thus confirm our current key finding that the [BF/S+L/Ep]-enhanced DC trafficking ability appears to be readily distinguishable from the LPS-induced DC mobility.

## Discussion

By using a combination of genomic and proteomic experimental approaches, we investigated the *ex vivo *effects of a candidate medicinal plant extract, [BF/S+L/Ep], on the differentiation and modulation activities of mouse BMDCs. This study extends our previous report [9,10], where human DCs were used in a similar functional genomics study. In addition to the *ex vivo *test system, this study also examined the *in vivo *effect of [BF/S+L/Ep] in experimental mice. Together, our current and previous findings on both human and mouse DCs, under *in vitro *as well as *in vivo *conditions, have provided us with a comprehensive information base for future translational research into potential clinical application of [BF/S+L/Ep] as botanical drugs or nutritional supplements. As demonstrated in Fig 2, our DNA microarray test systems and the experimental cell culture replicate samples were highly reproducible and consistent. We identified that close to 0.36% of the genes from the BMDC genome are significantly affected by treatment with [BF/S+L/Ep]. These differentially expressed and responsive genes were mostly related to cell adhesion and motility, immune response, signaling molecules and specialty enzymes. Some of these genes have been previously reported to be related to functions of DCs, but a large portion are reported here for the first time.

In this study, flow cytometry analysis on a number of CD markers showed that expression of several mouse DC surface markers was not significantly affected by treatment with [BF/S+L/Ep], this finding was not consistent with our previous data for human DCs [10]. This result may suggest that specific biochemical and cellular effects of herbal extract on DC maturation may vary considerably between different mammalian species, implying that future translation of experimental findings or results from animal models into human application need to be carefully addressed and considered with multiple references.

Immature DCs migrate from the blood to other tissues where they take up and process target antigens. Such DCs then subsequently migrate to the draining lymphoid tissues, resulting in the priming of naïve T cells following antigen presentation. During their migration, DCs are known to be involved in several adhesion or cognition events. For instance, E-cadherin, which is uniquely expressed by Langerhans DCs (LCs), permits the residence of LCs in the epidermis [24]. Such antigen encounter can result in down-regulation of E-cadherin that allows LC migration out of the skin [25]. The release of collagenase by DCs can facilitate their migration through the basement membranes [26]. Our data show that the gene expressing E-cadherin was down-regulated in DCs at both 4 h and 12 h after treatment with [BF/S+L/Ep]. Genes encoding for the cell adhesion protein integrin alpha 6 *(Itga6) *and the cell signaling protein gap junction membrane channel protein α1 *(Gja1) *were down-regulated at 4 h and 12 h post treatment, respectively. Moreover, expression of specific collagenases and proteases, such as *Mmp-8 *(Table 2), *Mmp-2*, and *Mmp-14*, and the macrophage elastase *Mmp-12 *(data not shown in Table 2 and 3, due to relatively low level slightly lower than 2 fold in fold change) were appearently up-regulated after 12 h of treatment. Taking these results together, and in conjunction with the *in vivo *trafficking assay data (Figure 5), we suggest that [BF/S+L/Ep] phytoextract can confer a modulatory effect on cell mobility of mouse BMDCs. In future studies it will be important and interesting to determine whether such an *in vivo *effect is also observed for human DCs, and thus for potential clinical application.

In general, various major inflammatory and immunomodulatory cytokines such as IL-1, TNF-α, IFNγ, IL-4, IL-5, IL-6, IL-13, and IL-17, which are readily induced by injury or infection, are known to stimulate the production of a spectrum of different chemokines though mediation via their respective receptors [27-29]. For instance, it is known that mice deficient in CCR2, the only known CCL2 receptor, have impaired Th_1 _responses [30], owing to a reduction of monocyte trafficking to the sites of inflammation [31]. In this study we show that a number of immune-related genes were up-regulated after 4 h and 12 h treatment of [BF/S+L/Ep]. These include genes encoding angiomotin (*Amot*), which is involved in cell movement and embryogenesis [32], chemokine (C-X-C motif) ligand 2 (*Cxcl5*), pro-platelet basic protein (*Cxcl7*), involved in chemokine activity and leukocyte transendothelial migration [33], immunoresponsive gene 1 (*Irg1*) and cytotoxic T-lymphocyte-associated protein 4 (*Ctla4*). These results suggest that [BF/S+L/Ep] may modify the chemotactic behavior of mouse DCs. Since some responsive chemokines such as CXCL5 and CXCL7, which we detected here in test DCs, are currently not well defined in terms of cellular function and biochemistry, further studies are needed to examine how [BF/S+L/Ep] can exert an effect on the secretion of these chemokines and to find out whether these chemokines do play a role in the chemoattraction and trafficking of treated DCs in the mouse system. On the other hand, our in vitro study did allow us to show that phenotypic expression of chemotaxis-related receptor proteins, including CCR5 and CCR7, can be significantly regulated in test mouse DCs treated with [BF/S+L/Ep] (Figure 6). Whether or not specific chemotaxis-related receptors, such as CCR5, can mechanistically modulate the [BF/S+L/Ep]-enhanced DC mobility will also require further investigations. Unlike our previous findings on human DCs [9], [BF/S+L/Ep] treatment had a readily distinguishable and a slower level effect on the expression of immune-related genes in mouse BMDCs. These results again suggest, like the effects observed for cell surface marker proteins, the immunomodulatory effects of phytocompounds from *Echinacea *extracts apparently could also differ substantially between humans and mice.

It is well known that DCs are the principal antigen presenting cells (APCs) for T-cell priming [34]. It is important to note, however, that DCs are also involved in the induction of central and peripheral tolerance [35]. One protein, called cytotoxic T lymphocyte-associated antigen 4 (CTLA-4), is known as an immunomodulatory membrane molecule. By decreasing T-cell responsiveness and raising the threshold for T-cell activation, CTLA-4 plays a critical role in the maintenance of peripheral T-cell tolerance [36,37]. Here, we found that the expression of *Ctla4 *was drastically up-regulated in DCs after 12 h of treatment with [BF/S+L/Ep]. Whether the CTLA-4 signaling system for DCs were also affected by our test phyto-compound and could in turn further affect T cell or other DC associated immunities would need to be further analyzed in future studies.

In human DCs, we showed that [BF/S+L/Ep] can effect the expression of specific cytoskeleton proteins, including macrophage-capping protein, cofilin, profilin, F-actin capping protein β subunit, and laminin A/C [10]. In the present study using mouse DC cells, our 2D gel electrophoretic analyses on DC proteins revealed that expression of several cytoskeleton-related proteins, such as alpha-actin-2 and F-actin capping protein alpha and macrophage capping protein, were also altered by [BF/S+L/Ep]. These results together suggest that Ep phytocompound extract modulates specific cytoskeleton functions, which are known to play a role in testing DCs to specific immune processes. A group of specific proteins, such as annexin A4, which can reversibly modify various membrane properties (e.g., fluidity and permeability), anchoring of cytoskeletal elements, aggregation of vesicles, and the regulation of ion conductance [38], and peroxiredoxin 4, which exhibits thioredoxin-dependent peroxidase activity and regulates the activation of the transcription factor NF-κB [39], were up-regulated in mouse DCs after treatment with [BF/S+L/Ep] (Figure 4B). These results suggest again that [BF/S+L/Ep] may affect the cell mobility and NF-κB signaling of BMDCs. Moreover, expression of several cellular metabolism-related proteins, including vimentin, proliferin-1 precursor-1, -2, -3, phenylalanyl-tRNA synthetase, phosphoglycerate mutase 1, cathepsin S, nucleoside diphosphate kinase and alpha-enolase were also affecteded by [BF/S+L/Ep] treatment (Table 4). Whether these changes are functionally related to specific cellular and molecular activities of mouse DCs needs to be further investigated.

Previous studies have demonstrated that specific alkylamides of *E. purpurea *can effectively stimulate macrophage function in healthy rats or RAW 264.7 cells [40,41]. In addition, specific *in vitro *effects of *Echinacea *extract and several of its phytochemical components on NF-κB expression in Jurkat cells (a human T-cell line) have been reported [42]. Recently we reported that phytocompounds from *E. purpurea *extracts could affect specific cell-surface marker expressions and cytoskeleton rearrangement in treated human DCs [9,10]. These findings suggest that a spectrum of specific immunomodulatory effects in DCs can be modulated by phytochemicals from *E. purpurea *at the cellular level. It is interesting to find that in this study, although the specific groups of responsive RNA transcripts and proteins seem to differ substantially from the results obtained from our previous studies on human DCs, the end result in terms of cellular functions seems to suggest that many of them, such as cytoskeleton-associated gene or protein expression can in fact be quite similar, and this provides valuable correlated information for future evaluations of the various molecular mechanisms of these effects. The *in vivo *DC trafficking results obtained in this study may further suggest the importance of the mobility and cytoskeletal components of the differentially expressed transcriptome for future evaluation of pharmaco-genomics activities in a mouse DC system *in vivo*. Besides, we believe that our current transient labeling system for DCs may provide future application to translational research on phytochemical or herbal medicinal effect on DCs.

## Conclusion

In this study, data obtained from both *in vitro *transcriptomic analyses and *in vivo *DC trafficking assays suggested that [BF/S+L/Ep] treatment can affect DC mobility. The effects of this bioactive herbal extract on expression of cytoskeleton-related RNA transcripts as well as on differential protein expressions related to cell adherence and other DC functions add complementary and supportive information to our previous studies on human DCs [9,10]. Taken together, our research shows that this candidate herbal medicine may cause drastic modulatory effects on specific immune cells (DCs) by regulating key cellular behaviors. In addition, bioinformatics studies from this and our previous study [10] have revealed a group of candidate target molecules and signaling networks, for example the JNK, PP2C-α, AKT and MAPKAPK pathways, that we believe warrant future systematic studies as key targets for immunomodulatory phytomedicines, via mediation with dendritic cells.

## Methods

### Plant materials and plant extract preparations and fractionations

*Echinacea purpurea *plants grown to the flowering stages were harvested from a reputable organic farm in Puli, Nantou County, Taiwan as previously described [9]. Stem and leaf tissues of fresh plants were extracted at room temperature by imbibition in 70% aqueous ethanol for 35 days, mimicking a traditional Chinese medicine (TCM) preparation protocol. The final 70% ethanol-soluble fraction was dried under vacuum, re-suspended and dissolved in 1 liter of water and then successively partitioned with ethyl acetate (1 L × 3 times) and *n*-butanol (1 L × 3 times) to yield three subfractions designated as the EA, BuOH and water (H_2_O) fractions of the (S+L) extract [10,43]. The percentage yield for the BuOH fraction of the stem and leaf tissue extract [BF/S+L/Ep] was 7.23% of the 70% ethanol extracts by dry weight.

### Culture of DCs from bone marrow

DCs were generated by using the method described by Inaba *et al*. [44]. Six-week-old female BALB/c (H-2Kd, I-Ad) mice were purchased from the National Laboratory Animal Center and kept under specific pathogen free (SPF) conditions. Femurs and tibiae were removed after euthanasia and the bone marrow was flushed with RPMI-1640 medium using a syringe with a 0.45-mm needle. Red blood cells in suspension were lysed with ACK lysing buffer (150 mM NH_4_Cl, 1.0 mM KHCO_3_, 0.1 mM EDTA) for 5 min. Bone marrow cells were suspended at concentration of 1×10^7^cells/30 ml complete media (CM, RPMI-1640 supplemented with 10% fetal bovine serum (FBS), 2 mM L-glutamine, 1% of nonessential amino acids and 100 U/mL penicillin and 100 μg/mL streptomycin). Test cells were cultured for up to 9 days with 1000 U/mL of GM-CSF at 37°C, 5% CO_2_. On day 3, two-thirds of the medium was removed and 30 mL fresh medium with GM-CSF was added to the cells. On day 6, culture plates were gently swirled and the floating and loosely adherent cells were discarded. Aliquots of 75% of culture media were replaced with fresh culture medium with GM-CSF. On day 9, non-adherent cells were collected and used as the immature DC population for subsequent tests and analyses.

### Treatment of DCs with E. purpurea extract

[BF/S+L/Ep] was dissolved in 100% pure and endotoxin-free dimethyl sulfoxide (DMSO). The working concentrations of each extract sample were prepared by serial dilutions to 75 μg/mL. This dosage for stimulation of DCs was chosen based on our results obtained by MTT assay: treatment of test cells with [BF/S+L/Ep] at 0, 25, 50, 75, 100, 500 μg/ml resulted in a cell viability, on average, of 100%, 102%, 107%, 104%, 99% and 73%, respectively, as compared to the vehicle control (data not shown). Treatment at 100 μg/ml [BF/S+L/Ep] might cause some limited cytotoxicity, whereas the dosage of 75 μg/ml does not result in any cytotoxic effects. The final DMSO concentration reached 0.1% in each DC culture. An aliquot of a 0.1% DMSO solution in medium only was thus used as a vehicle (negative) control. LPS from *Escherichia coli *(serotype 055:B5) was purchased from Sigma (St. Louis, MO) and used for cell activation or quality control at 1 μg/mL.

### Flow cytometric analysis

All cell samples were stained using a direct immunofluorescence staining method. For each step of the staining, 2 × 10^5 ^cells were treated with specific antibodies for 30 min at 4°C in 45 μL of phosphate-buffered saline (PBS) containing 2% bovine serum albumin (BSA). Test cells of each experimental group were first incubated with purified anti-CD16/CD32 antibody (mouse IgG2a, 93) for 10 minutes on ice. Fluorescein isothiocyanate (FITC)- or phycoerythin (PE)-labeled monoclonal antibodies were then used for staining of MHC class II (I-Ab, mouse IgG2a, 2G9), CCR4 (mouse IgG, 2G12), CCR5 (mouse IgG, HM-CCR5), CCR7 (mouse IgG2a, 4B12), CD40 (mouse IgG, HM40-3), CD80 (mouse IgM, 16-10A1), CD86 (mouse IgG, GL-1) and CD11c (mouse IgG, N418) on test cells. All antibodies were purchased from Biolegend (San Diego, CA). After cell samples were incubated with test antibody at 4°C for 40 min, cells were washed twice with PBS and fixed with 1% paraformaldehyde. Test cells were then analyzed in a Coulter EPICS XL flow cytometer (Beckman/Coulter).

### DNA microarray analysis for gene expression

Total RNA was isolated using TRIzol (Invitrogen) according to the manufacturer's instruction and used to generate cRNA targets. A total of 7 μg of RNA from each sample was used to synthesize the first strand cDNA using T_7_-Oligo (dT) primer and T7 RNA polymerase by *in vitro *transcription (IVT) reaction. The biotinylated cRNA products were then cleaned up according to the Affymetrix protocol. An aliquot of 15 μg of RNA per sample was then hybridized to a Affymetrix gene chip, the mouse Genome 430 2.0 array, containing 45,101 probesets and variants from over 34,000 well-characterized mouse genes, using a standard protocol suggested by the Affymetrix menu. Images of the array signals were collected on Affymetrix scanners. A total of 8 hybridizations were performed for immature DCs, while each time point and treatment was analyzed in duplicates on separate chips. Standard Pearson correlation coefficients were used to determine the consistency of gene expression in each replicate Affymetrix array.

$$\text{Pearson correlation value}=\frac{\sum \left(x-\bar{x})(y-\bar{y}\right)}{\sqrt{\sum {\left(x-\bar{x}\right)}^{2}\sum {\left(y-\bar{y}\right)}^{2}}}$$

Data was analyzed using Spotfire software, which includes algorithms that determine whether a gene is absent or present and whether the expression level of a gene in an experimental sample is significantly increased or decreased relative to a control sample. Changes in expression levels are presented as averages of log_2 _(BF/S+L/Ep treatment)/(DMSO treatment). The microarray data have been deposited to the Gene Expression Omnibus database at NCBI (GEO; http://www.ncbi.nlm.nih.gov/projects/geo/) under the accession number GSE19369.

### 2-D gel electrophoresis and image analysis

Total cellular proteins of immature dendritic cells were prepared using 0.3 mL of sample buffer [7 M urea (Bio-Rad), 2 M thiourea, 4% CHAPS (Sigma), 10% 1,4-dithioerythitol (Merck, Frankfurt, Germany), 2% Phamalyte 3-10] by vortexing for 1 h and then collecting the protein supernatants by 55,000 RPM and for 1 h. Protein concentration was determined by using a protein assay kit (Bio-Rad, Hercules, CA). 2-D gel electrophoresis was carried out using the Bio-Rad Protean IEF cell and Protean electrophoresis cell system as described previously [10,45] with modifications. Digitalized gel images were analyzed with 2-D analysis software (PD Quest, Bio-Rad) as previously described [10]. The image with the highest number of spots was selected as the master gel. Automatically detected images of protein spots in test gels were then manually edited to include the low intensity spots and correct for spot artifacts. For the match set containing images with pI 5-8, the spot volume (intensity integrated over the spot area) was normalized by the volume of total spots in the gel. The data was then exported to Microsoft Excel.

### Protein identification by MALDI-TOF-MS

Protein identification was performed as described previously [10]. Briefly, each gel slice was cut into small pieces with a scalpel. Reduction was achieved with 10 mM DTT at 57°C for 1 h. Alkylation reaction was performed with 55 mM iodoacetamide for 1 h at room temperature in the dark. Gel spots were then washed for 10 min alternately with 25 mM NH_4_HCO_3 _and acetonitrile. Gel pieces were completely dried using a Speed Vac. The dried gel pieces were then immersed in three volumes of trypsin solution (V5111; Promega, Madison, WI), at 20 ng/mL in 25 mM NH_4_HCO_3 _(freshly diluted). In-gel digestion was performed at 37°C overnight. The tryptic peptides were extracted from test gel pieces in 5 mL of 70% acetonitrile 5% HCOOH by sonication. The supernatant was dried under a Speed Vac and 6 μL of 1% HCOOH was then added to each test sample. Protein identification using MALDI-Q-TOF-MS analysis was performed by the Proteomics Core Facility of the Institute of Biological Chemistry, Academia Sinica, Taiwan [10]. The MS data with monoisotopic peptide masses were searched against the NCBI protein database using the MASCOT search engine (Matrix Science, London, UK).

### DC trafficking in vivo

To analyze the migration of *ex-vivo *cultured DCs under *in vivo *experimental conditions in test mice, 1 × 10^6 ^cultured DCs were labeled with fluorescein isothiocyanate isomer I (See Figure 5; green fluorescence dye) at 300 μg/mL at 37°C for 20 min, cells were then washed three times with PBS and subsequently injected into the tail vein of syngeneic BALB/c mice. Fresh lymph node (LN), spleen and liver tissues were harvested and frozen tissue sections of 12 μm in thickness were mounted on precleaned microscope slides (Superfrost/Plus; Fisher Scientific, Pittsburgh, PA), and stored at -80°C. To retain the fluorescent signal, the tissue sections were pretreated with 4% paraformaldehyde at room temperature. Tissue sections were first incubated with blocking solution (2% fetal calf serum in PBS) for 10 min. Nuclear staining was then performed with 4,6-diamidino-2-phenylindole (DAPI) and followed by washing with 1 × PBS (2 × 5 min each). Tissue sections were then mounted with 50% glycerol in H_2_O with coverslips. Fluorescence microscopy evaluation of immunostained frozen sections was performed using a Zeiss Axiovert 200 M microscope (Carl Zeiss, Heidelberg, Germany). Microscopy photos and images were captured with a digital camera (Orca ER; Hamamatsu) and processed using Axiovision 4.6.3 (Carl Zeiss). The number of individually florescent spot as test cells was then scored for comparative analysis and the data was exported to Microsoft Excel.

### Pathway analysis of representative genes involved in/associated with effects of [BF/S+L/Ep] on BMDCs

Using a Web-based entry tool developed by Ingenuity Systems [46], findings presented in peer-reviewed scientific publications were systematically encoded into ontology by content and modeling experts. A molecular network of direct physical, transcriptional and enzymatic interactions was observed between mammalian orthologs. For a better understanding of the temporal response of gene expression in the immune system, we constructed a prototypical cell showing the possible candidate signaling pathways, containing 35 genes (> 2-fold change) responding to treatment with [BF/S+L/Ep]. The candidate genes of interest also were keyed into TRANSPATH professional database, version 7.1, (Biobase Biological Databases GmbH, Germany) to identify possible target molecules and signaling pathways, using four hierarchical levels of regulation [9,10].

## Authors' contributions

SYY served as the key experimenter and author of the draft manuscript. WHW and KA provided useful revision and reorganization of the manuscript. BXW helped the *in vivo *DC trafficking experiments. HMW helped and introduced bioinformatics analyses on DNA microarray data as a team. NSY is PI and principal author of the manuscript. All authors read and approved the final manuscript
